# Give People What They Want, Feedback: Older Adult Perceptions on Cognitive Training Features to Increase Engagement

**DOI:** 10.1093/geroni/igaa057.1325

**Published:** 2020-12-16

**Authors:** Nelson Roque, Erin Harrell

**Affiliations:** 1 Pennsylvania State University, University Park, Pennsylvania, United States; 2 University of Alabama, Tuscaloosa, Tuscaloosa, Alabama, United States

## Abstract

According to Temporal Self-Regulation Theory (TST; Hall & Fong, 2007), adherence motivation can be driven by both positive and negative emotional reactions in which adherence is viewed in relation to gains versus losses. In a sample of 100 older adults (ages 64+), we explored participant-provided feedback related to intervention and game elements participants perceived would increase their adherence across five domains: (1) ability to adjust difficulty; (2) ability to change game design (from the preprogrammed American Western theme); (3) cognitive performance feedback; (4) ability to unlock extra game features (e.g., more levels); and (5) other elements. Ranked in order of frequency of endorsement, 53% endorsed: cognitive performance feedback; 47%: ability to adjust difficulty; 28%: ability to unlock new features; 27%: the ‘Other’ option; and 20% endorsed changing the game’s theme. This work has implications for models of adherence, specifically, the role that expectations of later cognitive feedback might play.

